# SCUBE3 downregulation modulates hepatocellular carcinoma by inhibiting CCNE1 via TGFβ/PI3K/AKT/GSK3β pathway

**DOI:** 10.1186/s12935-021-02402-z

**Published:** 2022-01-03

**Authors:** Pan Xu, Aoran Luo, Chuan Xiong, Hong Ren, Liang Yan, Qiang Luo

**Affiliations:** 1 Key Laboratory of Molecular Biology for Infectious Diseases (Ministry of Education), Institute for Viral Hepatitis, Department of Infectious Diseases, The Second Affiliated Hospital, Chongqing Medical University, Chongqing, People’s Republic of China; 2 Biotechnology and Nuclear Technology Research Institute, Sichuan Academy of Agricultural Sciences, Chengdu, 610061 People’s Republic of China; 3 Clinical Laboratory Department, Chongqing Hygeia Cancer Hospital, 200 SiXian Road, Chongqing, 401332 People’s Republic of China

**Keywords:** Hepatocellular carcinoma, SCUBE3, AKT, CCNE1, Cell proliferation

## Abstract

**Objectives:**

We aimed to verify the role of signal peptide-CUB-EGF-like domain-containing protein3 (SCUBE3) in the hepatocellular carcinoma (HCC) progression.

**Methods:**

The role of SCUBE3 in HCC cell proliferation, apoptosis, and cell cycle in *vitro* were detected using MTT assay, colony formation assay, 5-ethynyl-2´-deoxyuridine assay (EDU), Celigo cell counting assay, Caspase3/7 activity assay, and flow cytometry. The effect of SCUBE3 on HCC cell proliferation in vivo was inspected by a xenograft tumour model in nude mice. The related mechanisms were further studied.

**Results:**

The level of SCUBE3 was upregulated in HCC tissues and cell lines. Knockdown of SCUBE3 inhibited proliferation, promoted apoptosis, and induced cell cycle arrest in HCC cell lines in vitro and in vivo. Screening of cell cycle-related proteins revealed that CCNL2, CDK6, CCNE1, and CCND1 exhibited a significantly different expression profile. We found that SCUBE3 may promote the proliferation of HCC cells by regulating CCNE1 expression. The pathway enrichment analysis showed that the TGFβ signalling pathway and the PI3K/AKT signalling pathway were significantly altered. Co-immunoprecipitation results showed that SCUBE3 binds to the TGFβRII receptor. SCUBE3 knockdown inhibited the PI3K/AKT signalling pathway and the phosphorylation of GSK3β to inhibit its kinase activity.

**Conclusions:**

SCUBE3 promotes HCC development by regulating CCNE1 via TGFβ/PI3K/AKT/GSK3β pathway. In addition, SCUBE3 may be a new molecular target for the clinical diagnosis and treatment of HCC.

**Supplementary Information:**

The online version contains supplementary material available at 10.1186/s12935-021-02402-z.

## Background

Hepatocellular carcinoma (HCC) is the sixth most prevalent cancer in men and the seventh most prevalent cancer in women. HCC has become the second leading cause of cancer mortality worldwide [1]. Its pathophysiology is complex and involves many factors [2]. These include genetic changes, abnormal expression of specific cellular proteins, mutations of tumour suppressor factors, and overexpression of oncogenes [3]. The occurrence of HCC is associated with many signaling pathways, such as the Wnt signaling pathway [4], MAPK signaling pathway [5], TGFβ signaling pathway, and NF-κB signaling pathway [6]. Among them, TGFβ signaling is involved in all stages of liver disease progression, from initial liver injury, inflammation, and fibrosis to cirrhosis and cancer [7]. In the normal liver, TGFβ promotes liver cell differentiation and liver cell regeneration during embryogenesis. However, elevated TGFβ caused by chronic liver injury can lead to the differentiation of stellate cells into myofibroblasts. This can then lead to the death of a large number of liver cells, thereby promoting liver fibrosis and later cirrhosis [8]. In the early stage of liver tumour igenesis, TGFβ is expressed, and excessive activation of TGFβ signaling will accelerate tumour progression [9, 10]. Therefore, targeting the TGFβ signaling pathway can inhibit Hepatocellular carcinoma. TGFβ acts on hepatocytes by binding to a single transmembrane type I and type II receptor (i.e. TbRI and TbRII) with serine-threonine kinase activity [11]. The type II receptor phosphorylates the specific serine and threonine residues of the type I receptor in the near-membrane glycine domain after ligand-induced heteromeric complex formation. Once type I receptors are activated, extracellular signals are transferred from the membrane to the membrane, and intracellular signaling occurs by phosphorylation of specific proteins, in which receptor-regulated SMAD proteins, SMAD2 and SMAD3, play an important role. After the type I receptor is activated, SMAD2 and SMAD3 are activated, which then bind to SMAD4 to form heteromers. These are transferred to the nucleus where they regulate specific gene transcription responses [10, 12]. This is the typical sequence of events in the classical TGFβ signaling pathway. TGFβ can also trigger other signaling pathways in non-classical pathways, including MAPK, PI3K/AKT, and rho-like GTPase signaling pathways [13]. These pathways modulate not only SMAD-dependent responses but also induce non-SMAD-dependent responses. While the binding of TGFβ to its receptor can trigger intracellular signaling pathways, SCUBE3 can also bind to TGFβ receptors to trigger signaling pathways that can be triggered by receptor heteromers of type I and type II receptors [14]. SCUBE3 is a glycoprotein secreted by cells, which was first identified in human umbilical vein endothelial cells. It consists of five motifs: an n-terminal signal peptide, an epidermal growth factor (EGF)-like repeat sequence, a spacer region, three cysteine-rich domains, a complement protein C1r/C1s, and domains of Uegf and Bmp1 (CUB) c-terminal [15]. SCUBE3 can be clipped to release the n-terminal EGF-like repeat sequences and c-terminal CUB domains [14]. Recently, SCUBE3 was found to be involved in cardiac hypertrophy in mice through TGFβ1-mediated transcriptional activation [16]. SCUBE3 also participates in mouse embryonic development [17]. It is involved in the regulation of angiogenesis in lung cancer and promotes metastasis and invasion by promoting epithelial-mesenchymal transformation [18]. SCUBE3 also promotes the proliferation of osteosarcoma cells and influences the prognosis of patients with osteosarcoma [19, 20].

According to a recent study, a new asterosaponin (CN-3) isolated from the starfish Culcita novaeguineae led to the arrest of U251 cells in G1/S via the SCUBE3/ Akt/p-Akt/p53/p21/p27/E2F1 pathway [21]. Another interesting study showed that SCUBE3 acts as a BMP2/BMP4 co-receptor that recruits the BMP receptor complexes into raft microdomains, and positively modulates signaling possibly by augmenting the specific interactions between BMPs and BMP type I receptors. Yuh-Charn et al. Link SCUBE3 to processes controlling growth, morphogenesis, and bone and teeth development through modulation of BMP signaling [22].

Currently, there is no report on the role of SCUBE3 in HCC and its potent mechanism in HCC. Whether it plays a role in the proliferation, metastasis, and invasion of HCC is also not known. In this study, the function of SCUBE3 in HCC was investigated in vitro and in vivo.Knockdown of SCUBE3 inhibited proliferation, promoted apoptosis, and induced cell cycle arrest in HCC cell lines. We found that SCUBE3 may promote the proliferation of HCC cells by regulating CCNE1 expression. The pathway enrichment analysis showed that the TGFβ signaling pathway and the PI3K/AKT signaling pathway were significantly altered. Co-immunoprecipitation results showed that SCUBE3 binds to the TGFβRII receptor. SCUBE3 knockdown inhibited the PI3K/AKT signaling pathway and the phosphorylation of GSK3β to inhibit its kinase activity. Our research shows that SCUBE3 may be a potential target for the diagnosis and treatment of HCC.

## Materials and methods

### TCGA data analysis

Published data of mRNA expression in 114 normal liver tissues together with 1097 HCC specimens were downloaded from the TCGA database (http://ualcan.path.uab.edu/cgibin/TCGAExResultNew2.pl?genenam=SCUBE3&ctype=BRCA). The expression of SCUBE3 is shown in boxplots.

### Cell lines and culture

The HCC cell line Bel7404 was purchased from KeyGEN (Jiangsu, China). Human HCC cell lines Bel7404, HepG2, SMMC7721, Bel7402 and the normal liver cell line HL7702 were obtained from the Institute for Viral Hepatitis, the Key Laboratory of Molecular Biology for Infectious Diseases, Chongqing Medical University. Cells were cultured in high-glucose Dulbecc's modifed Eagl's medium'' (DMEM; Gibco, USA) supplemented with 1% penicillin/streptomycin (Solarbio, China) and 10% fetal bovine serum (FBS, Gibco, USA) at 37 °C with 5% CO_2_.

### Transfection experiments

Three short hairpin RNA (shRNA) for SCUBE3 and CCNE1 lentiviral vector and control vector (CON313, CON008) were designed by Ji Kai, Inc. (Shanghai, China). Their target sequences are listed in Additional file. Three SCUBE3-specific shRNA were inserted into the hU6-MCS-CBh-gcGFP-IRES-puromycin vector (Additional file 1: Table S1). Three CCNE1-shRNA were inserted into the hU6-MCS-CMV-puromycin vector (Additional file 1: Table S2). To investigate the effect of CCNE1 overexpression on SCUBE3 knockdown HCC cell lines. CCNE1 (NCBI reference sequence: NM_001238) lentiviral vector with a puromycin selection marker was constructed (Ji Kai, Inc., Shanghai, China), and empty vectors (Ubi-MCS-CMV-EGFP, CON319) were used as controls. Cells grown to ~ 30% confluency were infected at a multiplicity of infection of 20 using 5 µg/mL polybrene to aid viral attachment. Cells were selected with 2 μg/mL puromycin for a minimum of 4 weeks to select stably transfected lines. After that, the transfection efficiency was verified by observing the expression of GEP by RT-PCR and western blotting.

### Flag-SCUBE3 transfection

Full-length SCUBE3 cDNA (NCBI reference sequence: NM_152753) was cloned into the vector Ubi-MCS-3FLAG-CBh-gcGFP-IRES-puromycin. The vectors were confirmed via full sequencing and transformed into *Escherichia coli* (DH5α, CB101-02, TIANGEN) for purification using the Plasmid Maxi kit (Qiagen, N3009, Hilden, Germany). To construct the SCUBE3 overexpression Bel7404 cell line, transfection was conducted using Invitrogen™ Lipofectamine™ 3000 Transfection Reagent (Cat. no. L3000008) and 10 μg of plasmid in 10 mm dishes when the cell culture reached > 90% confluence on the day of transfection. After incubation for 8 h in a serum-free medium, the medium was replaced with DMEM (10% FBS). RT-qPCR and western blotting were used for detection after 48 h of transfection.

### Cell proliferation assay

Cell viability was detected quantitatively by the MTT assay, which is based on the determination of the activity of live cells (GenView, Cat. No. JT343). Briefly, cells were seeded in 96-well plates at 5 × 10^3^ per well in a final volume of 100 µL. After culturing for 24, 48, 72, 96, or 120 h, the number of viable cells was tested by incubating the cells with 50 μL MTT (5 mM) for another 4 h. The viability at each time point was represented as the optical density value, which was detected by using a Tecan Infinite plate reader (Cat. no. M2009PR) using an enzyme-linked immunosorbent assay plate reader at 450 nm.

### EdU incorporation assay

Cell proliferation was detected by using a Cell-Light EdU Apollo 555 In Vitro Kit (Cat. no. C10310, Beyotime Biotechnology, Shanghai, China) according to the manufaturer's protocol. In brief, different HCC cells were plated in 6-well plates. After transfection, the cells in each well were treated with EdU and incubated for 2 h, followed by washing twice with phosphate-buffered saline. Then, the cells were fed with 4% paraformaldehyde for 30 min at room temperature. Subsequently, the cells were treated with Apollo staining reaction liquid for another 30 min to detect positive cells. Finally, the cells were stained again with 4',6-diamidino-2-phenylindole (DAPI), and the immunofluorescence was observed using a fluorescence microscope. Cells were counted by a Cellometer (Auto1000, Nexcelom).

### Celigo cell counting assay

Lentivirus-infected Bel7404 cells were seeded into 96-well plates at a density of 2 × 10^3^ cells/well. The number of cells with green fluorescence in each scan plate was tested daily for 5 days by using a Celigo® Image Cytometer (Nexcelom, Lawrence, MA, USA) from the second day.

### Caspase 3/7 activity analysis and apoptosis assay

Cell apoptosis was detected by flow cytometry with an Annexin V/PI apoptosis kit (Cat. no. 559763, BD Biosciences, San Jose, CA, USA), according to the manufacturer's instructions. Cells were digested and centrifuged at 100 *rcf* at 4 °C for 5 min. Then, the cells were resuspended in 100 μL 1 × binding buffer and incubated with 5μL Annexin V-APC/PI in the dark at 25 °C for 15 min. The cells were suspended in 400 μL 1 × binding buffer to remove unbound moieties and subjected to flow cytometry (BD Biosciences, Oxford, UK). Bel7404 cells were incubated in a 96-well plate, and three blank wells were set up. Caspase 3/7 intracellular activity was detected by using the Apo-ONE® caspase 3/7 assay kit (Cat. no.G8091, Promega, Mannheim, Germany), and cell fluorescence intensity at 499 nm was measured by using Tecan Infinite plate reader. The experiments, including technical duplicates, were repeated at least three times.

#### Colony formation assays

A total of 500–800 cells were seeded into 60-mm dishes and cultured in DMEM for two weeks. The colonies were then fixed with 4% Paraformaldehyde and stained with a 0.5% crystal violet solution (KaiJi Biotechnology, China). The cells were washed several times with ddH2O and then dried. The digital camera was used to take pictures, and the clones were counted with Image J software. Each experiment was performed in triplicate and repeated three times.

#### Wound healing assay

Stably transfected cells were seeded into 6-well plates at a density of 1 × 10^6^/ml and cultured until confluent. A wound gap was scratched using a 1 mL pipette tip followed by three gentle washes with PBS to remove cellular debris, and cell migration was assessed following incubation in serum-free media for 48 h. Images were acquired using an inverted phase microscope (Ts2R, Nikon, Japan). The Wound healing rates were calculated using Image-J software and the following formula: the percentage of wound healing =  (the scratch area in 0–48 h)*100 /the area in 0 h.

#### Transwell assays

For migration assays, approximately 2 × 10^4^ Bel7404 cells suspended in 200 µL serum-free medium were seeded into the top chamber with 8.0 μm pores (Cat. no.MCEP24H48, BD Bioscience, USA). 700 µL 20% FBS DMEM was filled in the bottom of transwells. After 24 h of incubation, cells migrated through the membrane were fixed, stained, and counted under the light microscope. Each experiment was performed in triplicate.

### Reverse-transcriptase polymerase chain reaction (RT-PCR)

Total RNA in the cells was extracted using TRIzol® reagent (Cat. No.15596026, Life Technology, Carlsbad, USA) and reverse-transcribed into cDNA template using the ReverTra Ace qRT-PCR kit (Code No.FSQ-101, Toyobo, Japan), according to the manufacturer's instructions. The amplification parameters were set as follows: denaturation at 95 °C for 3 min, followed by 40 cycles at 95 °C for10 s, and at 60 °C for 30 s. The results were analyzed using CFX96 Manager Software (Bio-Rad, USA). The 2^−ΔΔCt^ method was used to evaluate the mRNA expression. The relative expression was calculated and normalized to GAPDH. The paired primers for each gene are listed in Additional file 1: Table S3.

### Affymetrix GeneChip analysis

The microarray analysis [23] was conducted by Affymetrix GeneChip analysis. The integrity of RNA was determined by an Agilent Bioanalyser2100 using Agilent RNA 6000 Nano Kit, and then the samples were prepared according to the Affymetrix GeneChipExpression Analysis Manual (Affymetrix). The arrays were washed and stained with streptavidin phycoerythrin. The staining intensities were determined using a GeneChip scanner 3000 (Affymetrix). The microarray transcriptional profiling was performed using GeneChip Operating Software (Affymetrix) at Gene Company Ltd. (Shanghai, China). The screening criteria for significantly different genes were: |Fold Change|> 1.5 and FDR < 0.05.

### Cell immunofluorescence and localization

Bel7404 and HepG2 cells were plated in 35 mm confocal culture dishes for 24 h. On the following day, the cells were treated as previously described [24]. Primary antibodies against SCUBE3 (ab189955, Abcam,1:50) and TGFβIIR (sc-17799, Santa cruz,1:50) were used at 1:50 dilution. After washing, the cells were then incubated with fluorescence-labelled secondary antibodies Alexa-Fluor 555 anti-mouse IgG and anti-rabbit IgG (1:200 dilution, Cell Signaling Technology, Danvers, MA, USA). Then, cells were washed and stained with DAPI. Finally, images of cells positive for SCUBE3 and TGFβIIR were captured using a confocal microscope (Nikon Corporation, Tokyo, Japan).

### Protein extraction and western blotting

Cells were lysed in a RIPA buffer (KeyGEN, Jiangsu, China) containing PMSF, a protease inhibitor, and a phosphatase inhibitor to collect total protein following the manufacturer's instructions. Membrane and cytoplasmic proteins of Bel7404 cells were cleaved with an extraction kit (KeyGEN, Cat no: KGP350-2/KGP3100-2, Jiangsu, China). The protein concentration was measured by using a BCA protein detection kit. Exactly 50 µg protein was loaded in each lane, followed by PAGE containing 10% SDS. After transferring to a 0.45 µm PVDF membrane (Merck Millipore, USA), the membrane was blocked in 5% bovine serum albumin (Cat#A8020, Solarbio Sciences, China) at room temperature for 2 h and incubated at 4 °C with primary antibodies overnight. The primary antibodies against human SCUBE3 (ab189955,1:1000) and TGFβIIR (ab186838,1:1000) were purchased from Abcam. Primary antibodies against CCND1 (#5550, 1:1000), CCNE1 (#20,808, 1:1000), CDK6 (#13,331, 1:1000), AKT (#4691, 1:1000), p-AKT (#4060, 1:1000), GAPDH (#5174, 1:1000), GSK3β (#5676, 1:1000), and p-GSK3β (#9322, 1:1000) were purchased from Cell Signaling Technology (USA). A mouse monoclonal antibody [FG4R] recognizing the DDDDK (FLAG) tag was purchased from Arigo Company (ARG62342, 1:2000). The membranes were washed with TBST buffer three times for 10 min and incubated with goat anti-rabbit (ab6721, Abcam, 1:10,000) or anti-mouse secondary antibodies (AS003, ABclonal, 1:6000). The target protein bands were visualized using enhanced chemiluminescence detection and processed using Image Lab software (Bio-Rad, USA). All western blotting analyses were performed at least three times.

### Co-immunoprecipitation assay

The cell lysates and Protein A/G Beads 4FF (Smart Lifesciences, China) were prepared according to the manufacture's instructions. The protein G beads are shipped and stored in 20% ethanol for preservation. The bead slurry consists typically of 50% beads, and these beads need to be washed before they are used to immunoprecipitate the immune complexes. 1 mg extracted protein was incubated with 2 µg DDDDK (FLAG) tag and TGFβIIR antibody overnight at 4 °C with gentle agitation, followed by 12 h incubation with 20 µl Protein A/G agarose beads at 4 °C with gentle agitation. Prior to incubation, the beads were suspended and washed three times with IP lysis buffer. The bead-antibody-antigen complex was then centrifuged at 800 rpm for 5 min at 4 °C, and the bead complex was washed three times with IP lysis buffer (the supernatant of the last collection as the input group samples). After immunoprecipitation of the immune complexes by protein G beads and eluting them, the co-immunoprecipitated proteins in the cell lysate and eluate of the co-immunoprecipitation sample were visualized by SDS-PAGE and western blotting. Caution was taken not to disturb the pelleted protein G beads when loading the samples for PAGE.

### Animal experiments

Female BALB/c nude mice (6 weeks old) were purchased from Ling Chang Biotechnology (Shanghai, China). The mice were randomly divided into two groups (n = 10). Briefly, 2 × 10^7^/mL shSCUBE3 Bel7404 cells or negative control Bel7404 cells were subcutaneously injected into the flanks of nude mice. The tumour volume was monitored every 3 days for 15 days by measuring the width and length of each tumour. Live animals were imaged in an IVIS 100 imaging system (Lumina LT, PerkinElmer) after using isoflurane anaesthesia on day 25. Then, the animals were euthanized with 2.5 L/min CO_2_ until breathing stopped (approximately 5–8 min)**,** and tumour samples were collected, and the weight of each tumour was determined. All the animals were treated according to the guidelines of the Institutional Animal Care and Use Committee of Chongqing Medical University.

### Statistical analysis

All statistical analyses were performed by using the SPSS statistical program (version 25.0, Chicago, IL, USA) and GraphPad Prism 8.0 (GraphPad Software, LaJolla, CA, USA). The significance between the two groups was tested using Student's t-test. Data are shown as the mean value and standard deviation. P-values < 0.05 were considered to indicate statistical significance.

## Results

### SCUBE3 is upregulated in HCC and is associated with poor prognosis

**Fig. 1:**
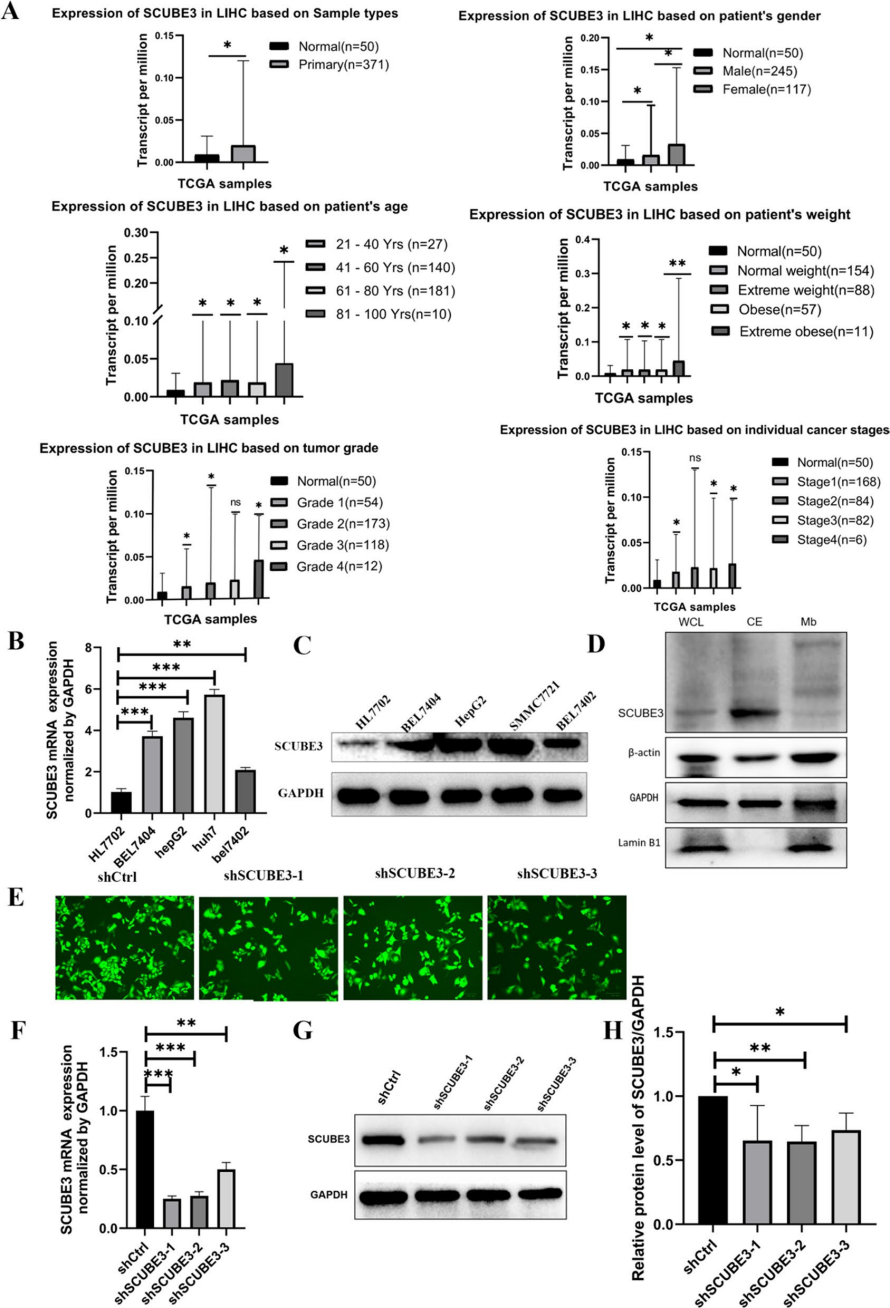
Expression analysis of SCUBE3 and establishment and validation of scube3 knockdown cell model. **A** SCUBE3 expression in tumour and normal tissues in the TCGA database from UALCAN database. SCUBE3 expression is based on the pa'tients sex, age, body weight, tumour grade and cancer stage. **B**, **C** RT-qPCR and western blot analysis of SCUBE3 mRNA and protein expression in normal hepatocytes HL7702 and four HCC cell lines (Bel7404, Bel7402, HepG2, and SMMC7721). **D** Western blotting was used to detect the expression level of SCUBE3 in the whole protein, cytoplasmic protein, and membrane protein samples of Bel7404 HCC cells. **E** Bel7404 cells were transfected with control shRNA (shCtrl) and three shSCUBE3 lentiviruses (shSCUBE3-1, shSCUBE3-2, shSCUBE3-3). Green fluorescence protein (GFP) expression illustrated to transfect with lentivirus successfully. **F** The expression levels of SCUBE3 in the cells were determined through reverse-transcriptase PCR. **G**–**H** The protein expression of SCUBE3 was examined by western blotting (*n* = 3, ns,no significance,*p < 0.05,**p < 0.01, and ***p < 0.001 compared with normal cells)

SCUBE3 expression in normal and cancer tissues was analyzed using The Cancer Genome Atlas (TCGA) liver HCC data on UALCAN (http://ualcan.path.uab.edu/cgibin/TCGAExResultNew2.pl?genenam=SCUBE3&ctype=LIHC). As shown in Fig. 1A, SCUBE3 expression in tumour tissue was upregulated compared with that in the adjacent normal tissue. Female sex, older age, and higher body weight correlated with a significantly higher expression of SCUBE3 in liver cancer tissues.

We first assessed the relative expression levels of SCUBE3 in four HCC cell lines, including Bel7404, Bel7402, HepG2, and SMMC7721 and one normal human hepatocyte line HL7702 by real-time PCR (Fig. 1B) and western blotting (Fig. 1C). SCUBE3 expression in HCC cells was significantly higher than that in normal hepatocytes. Bel7404 was selected for subsequent cell experiments for its relatively high SCUBE3 expression among the four HCC cell lines. In addition, we compared the expression levels of SCUBE3 in whole protein, cytoplasmic protein, and membrane protein samples of HCC cells by western blotting and found that SCUBE3 was mainly expressed in the cytoplasm (Fig. 1D). In all, we speculate that SCUBE3 may play an important role in HCC progression.

### Knockdown of SCUBE3 expression suppressed proliferation, promoted apoptosis, and induced cell cycle arrest in HCC cells

**Fig. 2:**
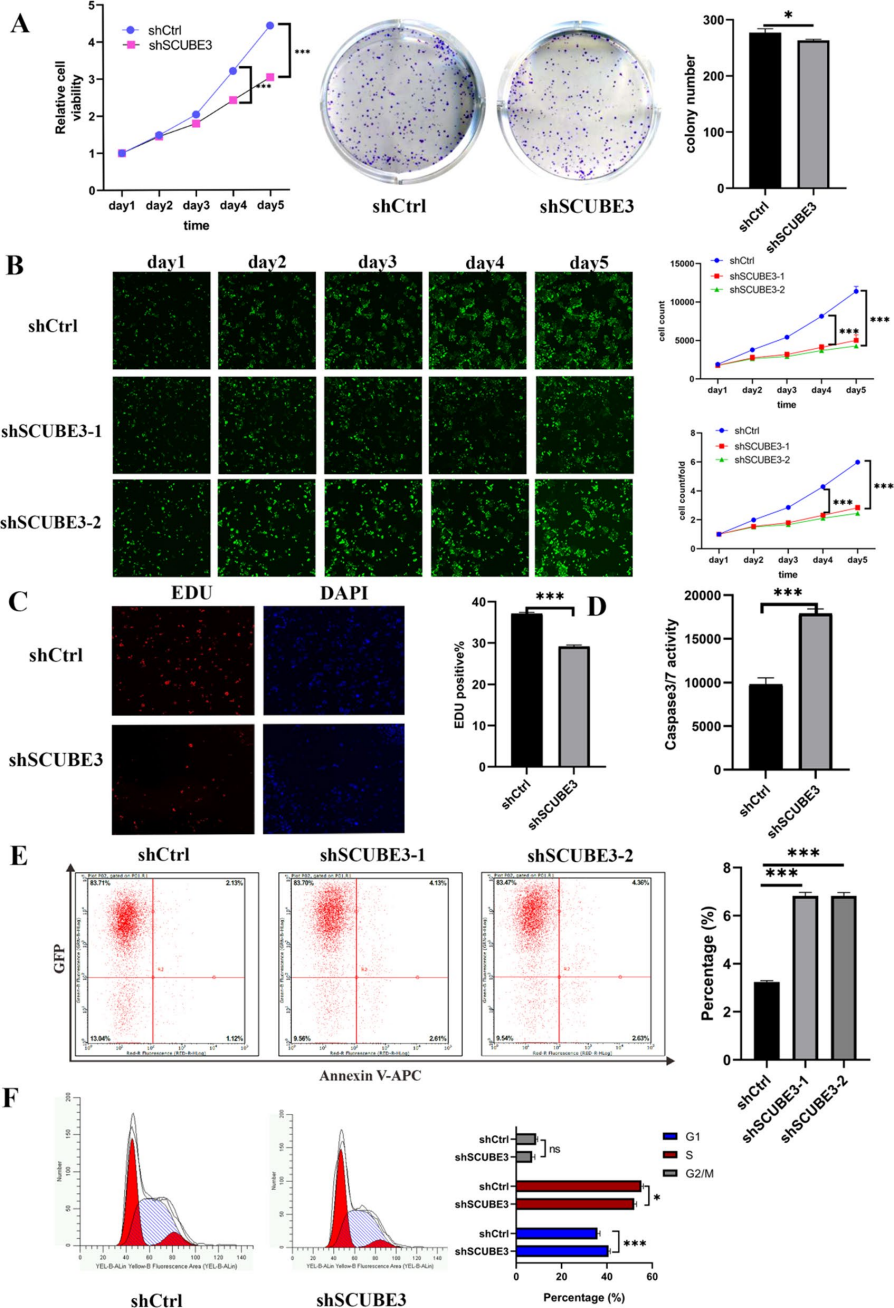
Knockdown of SCUBE3 expression suppressed proliferation, promoted apoptosis, and induced cell cycle arrest in HCC cells. **A** MTT-assays were performed to evaluate the viability of negative control and cells expressing low levels of SCUBE3, and Colony formation assay using knockdown of SCUBE3 cells. **B** The proliferation rate of Bel7404 cells was reduced by SCUBE3 knockdown, which was observed using the Celigo cell counting assay. **C** EdU assays were used to determine the effect of SCUBE3 on the proliferation of Bel7404 cells. **D** Relative caspase activity measured by caspase 3/7 assay showed apoptosis in knockdown of SCUBE3 cells (shSCUBE3). **E** Effect of SCUBE3 on apoptosis was analyzed through flow cytometry using Annexin V-APC staining. **F** Cell cycle distribution after inhibition of SCUBE3 was analyzed through flow cytometry (*n* = 3,*p < 0.05, **p < 0.01, ***p < 0.001. ns, no significance, KD, knockdown, NC, negative control)

To determine whether SCUBE3 plays a role in proliferation and apoptosis, we engineered lentivirus-transduced stable knockdown of SCUBE3 in Bel7404 cells by using three individual shRNAs (Fig. 1E–H). We found that knockdown of SCUBE3 expression was associated with inhibition of cancer cell proliferation MTT, colony formation assay and EdU assays were performed to investigate the effects of silencing of SCUBE3 on HCC cell proliferation in vitro (Fig. 2A, C). In addition, we revealed that the knockdown of SCUBE3 markedly suppressed the proliferation of Bel7404 cells using the Celigo cell counting assay (Fig. 2B). The effect of SCUBE3 on cell apoptosis and cell cycle arrest was further studied. The caspase-3 activity in the KD group was significantly higher than that in the NC group (Fig. 2D). We found that knockdown of SCUBE3 slightly increased the number of Annexin V-APC /PI-positive cells (Fig. 2E). In addition, we analyzed the cell cycle distribution. In Bel7404 cells, the knockdown of SCUBE3 significantly increased the number of cells in the G1 phase but reduced the number of cells in the S and G2/M phases (Fig. 2F). In a word, downregulation of SCUBE3 represses proliferation and promotes apoptosis of HCC cells.

### Knockdown of SCUBE3 suppresses tumour growth in vivo

**Fig. 3:**
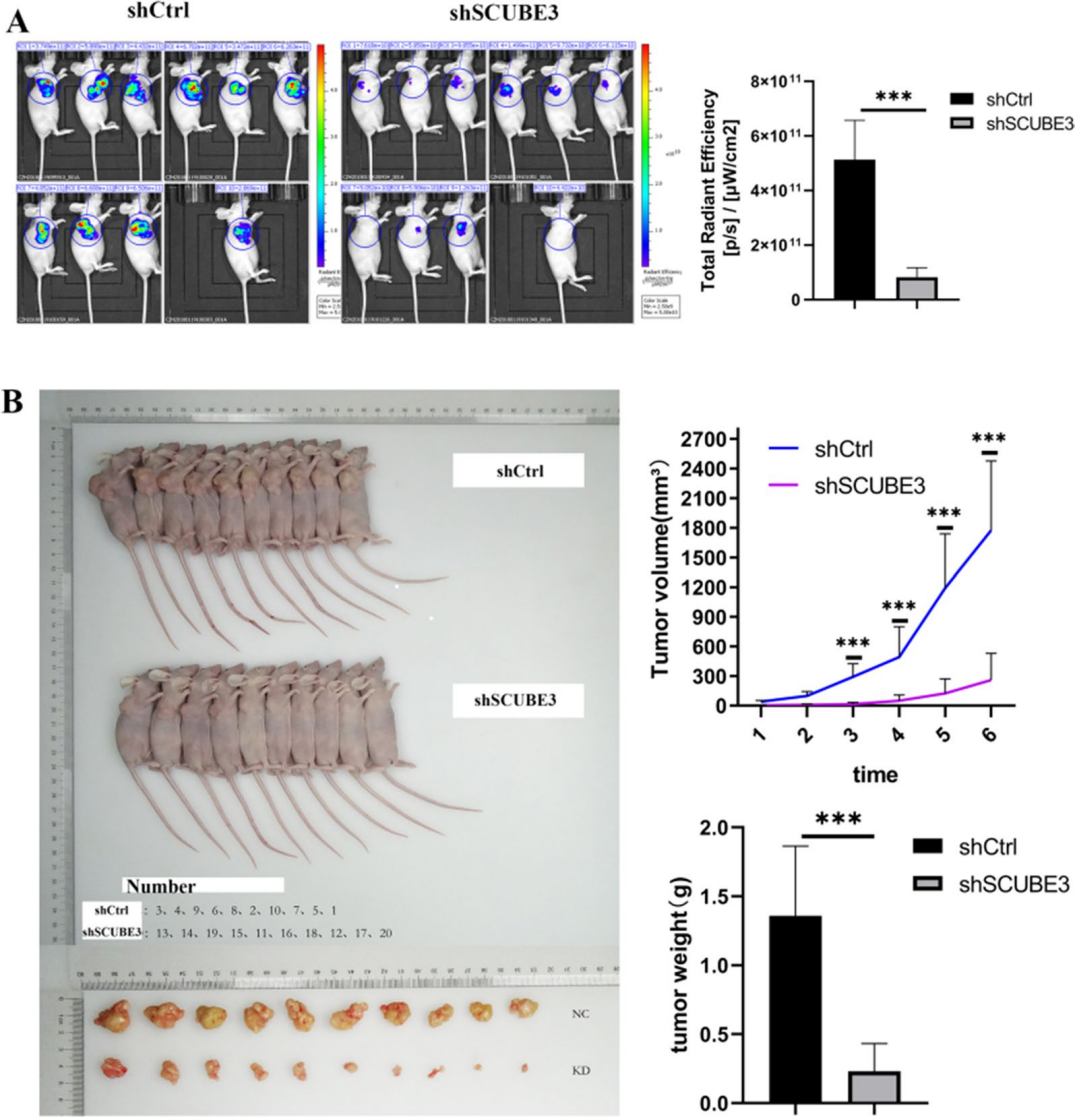
SCUBE3 promotes HCC cell proliferation in vivo. **A** In vivo imaging of subcutaneous tumour formation in nude mice implanted with SCUBE3 knockdown and control cells. Quantification of fluorescence values of the two groups. **B** Photographs of tumours taken after four weeks. Comparison of tumour volume and weight of SCUBE3 knockdown cells and control cells after subcutaneous tumour formation in nude mice (*n* = 10, The results represent the mean ± SD,***p < 0.001)

To evaluate the effects of SCUBE3 on tumour growth in vivo, Sh-control and ShSCUBE3 cells were injected into the armpits of nude mice (n = 10). The tumour-bearing mice were also photographed and analyzed by a non-invasive in vivo imaging system before sacrifice. The fluorescence signal for the region of interest was found to be significantly attenuated compared to that in control mice (transplanted with sh-control cells) (Fig. 3A). Moreover, the KD group's tumour weight and volume were significantly lower than those in the control group (Fig. 3B). These results indicated that knockdown of SCUBE3 decreases the proliferation of HCC cells in vivo.

### Effects of SCUBE3 depletion on the transcriptome of Hepatoma cells

**Fig. 4:**
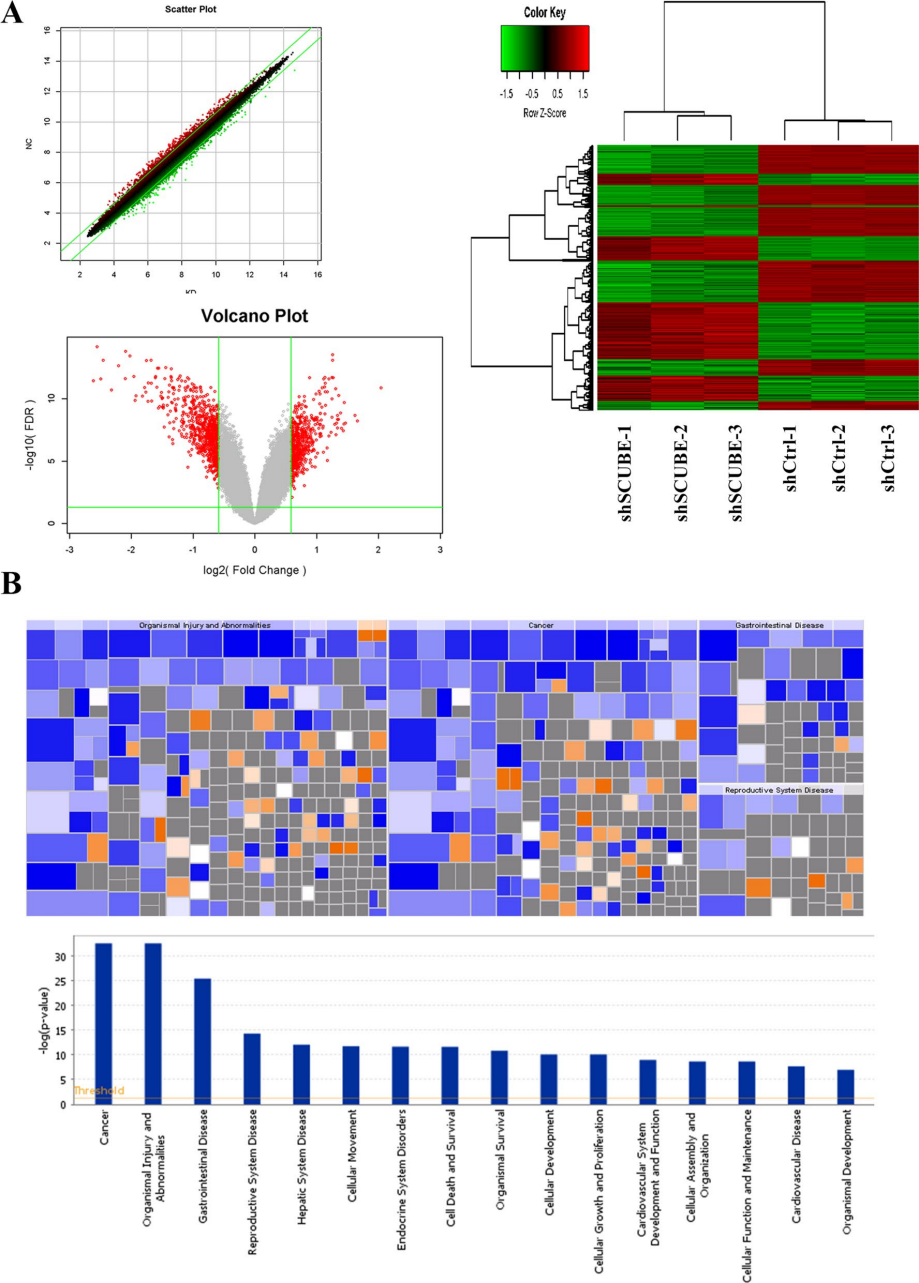

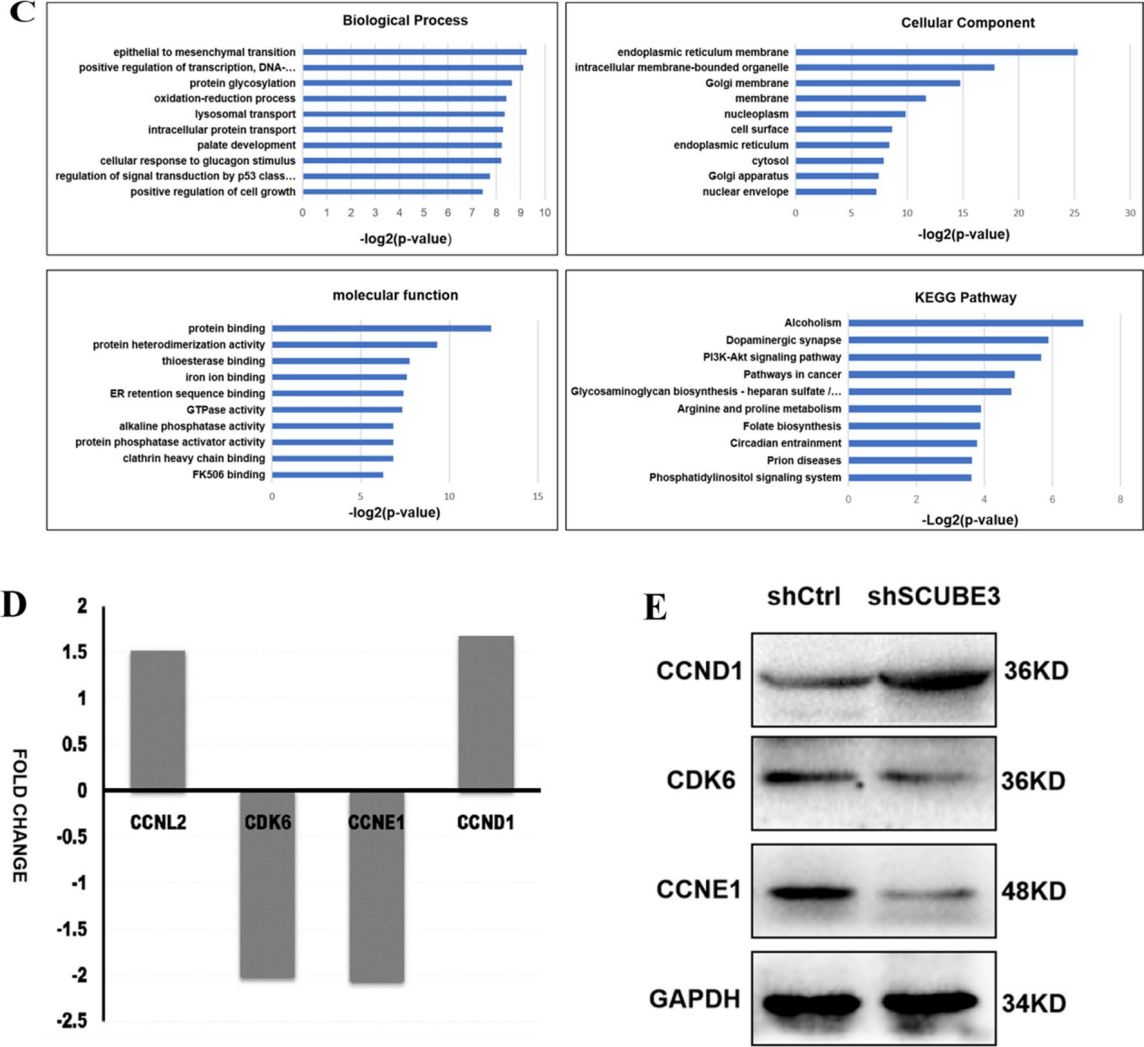
Affymetrix GeneChip microarray analysis and validation. **A** Analysis of significant differences according to the screening criteria of fold change |> 1.5| and false discovery rate (FDR) < 0.05. Scatter plot, volcano plot, heat map were constructed based on the genes differentially expressed between the control and SCUBE3-knockdown cells. **B** Disease and functional enrichment analysis of differential genes by IPA. The heat map of disease and function showed the relationship between the activation and inhibition of disease and function by the change of differential gene expression level. Orange indicates that the disease or functional state is activated (Z-score > 0), blue indicates that the disease or functional state is inhibited (Z-score < 0), and grey indicates that the disease or functional state is not determined (Z-score cannot be calculated). According to the internal algorithm and standard of IPA, Z-score > 2 means that the disease or function is significantly activated, and Z-score <—2 means that the disease or function is significantly inhibited. The diseases or functions significantly activated include Organic death (Z-score = 3.247), morbidity or mortality (Z-score = 3.200), etc.The diseases or functions significantly inhibited included: viral infection (Z-score = − 3.110), invasion of cells (Z-score = − 2.933). Histogram of disease and functional enrichment analysis statistics. **C** Significant GO terms associated with SCUBE3 knockdown in HCC cells.Analysis of cellular component (GO-CC), Analysis of biological process (GO-BP). Analysis of molecular function (GO-MF). KEGG Pathway enrichment. **D** Four genes associated with cell cycle regulation were screened by the microarray Chip analysis following the screening criteria. **E** The expression levels of three proteins (CCNE1, CCND1, CDK6) were verified by WB

To further investigate the molecular mechanism underlying the SCUBE3-mediated promotion of HCC cell proliferation, total RNA of cells was extracted to apply RNA-Seq analysis, and then the Affymetrix GeneChip microarray analysis was used to screen and identify differentially expressed genes (DEGs). The quality of the CHIP-seq data was initially assessed as shown in Additional file 1: Figure S1. According to DEGs painted into a heat map, scatter plot, and volcano plot, there were 405 upregulated genes and 489 downregulated genes in the SCUBE3-cells (Fig. 4A). These DEGs were analyzed for "Diseases and Bio-function"by ingenuity pathways analysis (IPA).The heat map of disease and function shows the relationship between the activation and inhibition of disease and function by a change in the differential gene expression level. The diseases or functions significantly activated in this study included: organic death, morbidity, and mortality. The diseases or functions significantly inhibited included: viral infection and invasion of cells, Cancer, organism damage and abnormality, gastrointestinal disease, reproductive system disease, liver system disease, cell movement, endocrine system disorder, cell death and survival, organism survival, and cell development were the top 10 diseases (Fig. 4B). The top three signaling pathways that were downregulated in KD were alcoholism, pathways in Cancer, and the PI3K-AKT signaling pathway. GO molecular function analysis showed that the majority of the corresponding proteins were annotated with a protein-binding function when considering both upregulated and downregulated genes. In the cellular component group, DEGs were mainly enriched in the cytosol, nucleoplasm, membrane, and extracellular exosome (Fig. 4C). The four significantly enriched differential genes related to cell cycle regulation were CCNL2, CDK6, CCNE1, and CCND1 (Fig. 4D). According to previous experimental results, SCUBE3 may promote cell proliferation by influencing the cell cycle. We verified the expression of three proteins related to cell cycle regulation by western blotting (Fig. 4E). The results were consistent with the results of Chip analysis and showed that the most significant change was CCNE1. Together, these findings demonstrated that the function of SCUBE3 to promote proliferation might depend on the presence of CCNE1 and PI3K-AKT signaling pathways.

### SCUBE3 promotes proliferation of hepatoma cells through CCNE1

**Fig. 5:**
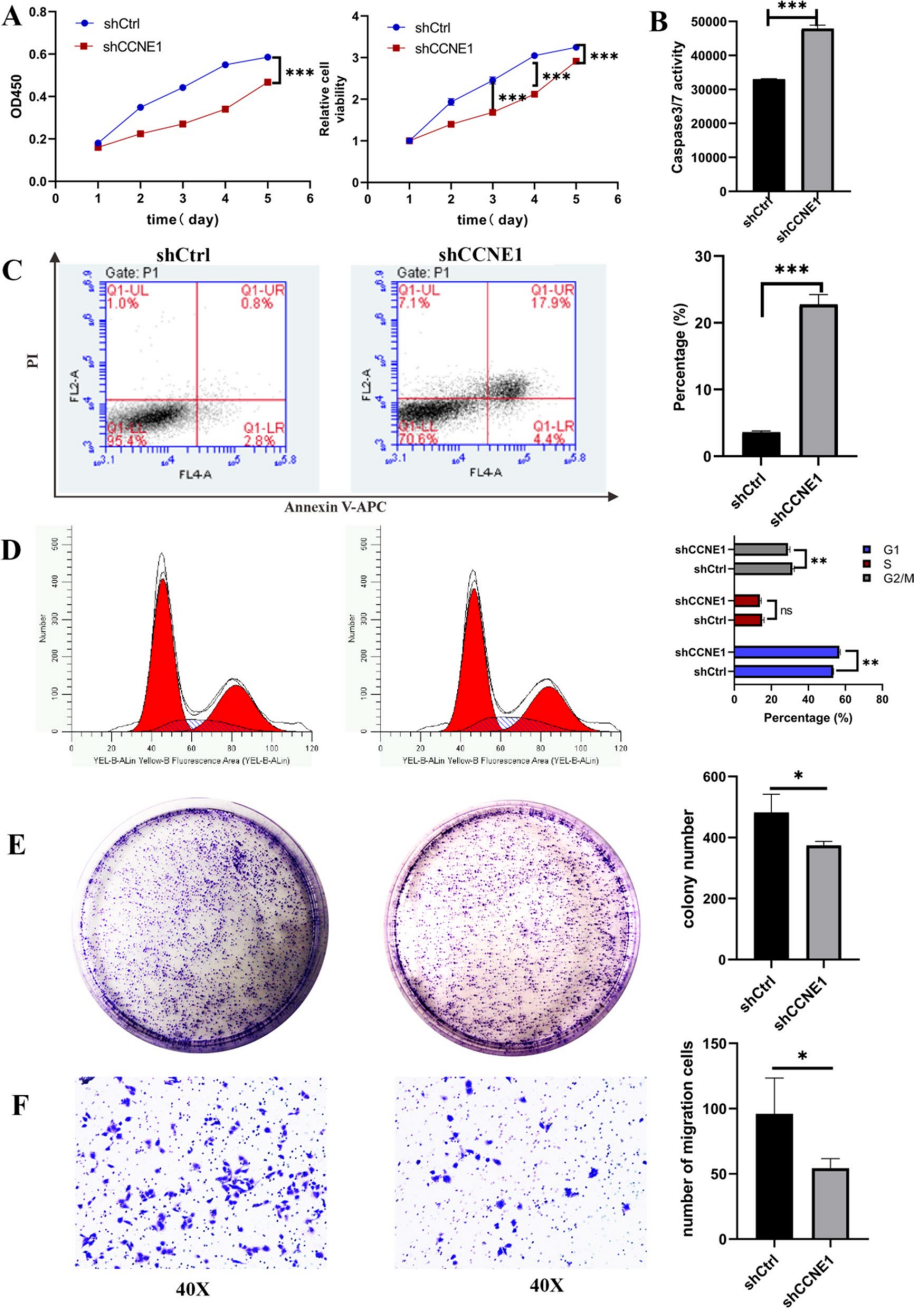
CCNE1 silence inhibit Bel7404 cell proliferation, migration, arrested G1/S transition and promote apoptosis. **A** 5 days of continuous MTT assays. **B** Knockdown of CCNE1 remarkably enhanced caspase-3/7 activity in the shCCNE1 group. **C** Knockdown of CCNE1 promoted cell apoptosis. **D** Analysis of cell cycle profiles after CCNE knockdown in Bel7404 cells. **E** Colony formation assay using cells infected with CCNE1 lentiviral vector and empty vectors. Representative images were acquired, and the colonies were counted after two weeks. **F** The inhibition effect of CCNE1 silence on cell migration was assessed by transwell assay. After 24 h of incubation, the cells were fixed and stained. Representative images of transwell chamber assays were acquired, and quantification of the number of cells which migrated through the basement membrane (*n* = 5 per group) (*p < 0.01, **p < 0.01,***p < 0.001 compared with shCtrl)

Therefore, we studied CCNE1 as the downstream effector of SCUBE3. First, the expression of CCNE1 in human liver cancer was analyzed by using TCGA data on UALCAN (http://ualcan.path.uab.edu/cgibin/TCGAExResultNew2.pl?genenam=CCNE1&ctype=LIHC), and the results showed that the expression of CCNE1 in human liver cancer tissues was significantly higher than that in adjacent normal tissues (Additional file 1: Figure S2A). The expression of CCNE1 was not correlated with the sex of the patients, i.e., CCNE1 expression in liver cancer tissues of female patients was not significantly different from that in liver cancer tissues of male patients (Additional file 1: Figure S2B). CCNE1 expression significantly differed in patients of different age groups, while the difference between CCNE1 expression in the liver cancer tissue and normal liver tissue was significant, CCNE1 expression in normal liver tissue did not differ between the different age groups (Additional file 1: Figure S2C). CCNE1 expression in liver cancer tissue differed significantly from that in normal liver tissue in the groups with different cancer stages (Additional file 1: Figure S2E), but CCNE1 expression in the liver cancer tissue differed significantly only between stages 2 and 4 and between stages 3 and 4 (Additional file 1: Figure S2F). Thus, CCNE1 expression has a particular relationship with the occurrence and development of liver cancer. We also analyzed the impact of CCNE1 expression on patient survival using TCGA human liver cancer data on UALCAN (http://ualcan.path.uab.edu/cgibin/TCGAsurvival1.pl?genenam=CCNE1&ctype=LIHC), Moreover, the results showed that the overall survival rate of liver cancer patients with a high expression of CCNE1 was significantly lower than that of liver cancer patients with a low expression of CCNE1 (Additional file 1: Figure S3). The relationship between CCNE1 expression and sex, body weight, and race, but not liver cancer grade, of liver cancer patients, significantly affected the overall survival rate of these patients. To verify the role of CCNE1 in the proliferation of HCC cells, we first knocked down CCNE1 in Bel7404 cells (Additional file 1: Figure S4) and conducted MTT, Colony formation assay, and EdU proliferation experiments. The cell proliferation in the KD group was significantly lower than that in the NC group (Fig. 5A, E). We examined the effect of CCNE1 on cell migration using transwell and wound healing assay. In the transwell invasion assay, the shCCNE1 groups were less migration than the shCtrl cell line (Fig. 5F). The wound healing capability of shCCNE1 cells was weaker at 48 h post-scratch than shCtrl cells (Additional file 1: Figure S6). Unfortunately, there was no significant statistical significance here. Next, apoptosis was detected by Annexin V/PI staining and by determining caspase 3/7 activity (Fig. 5B, C). The number of apoptotic cells in the KD group was significantly higher than that in the NC group. The caspase 3/7 activity of cells in the KD group was significantly higher than that of cells in the NC group. Finally, we assessed the effect of SCUBE3 on the cell cycle of liver cancer cells. The number of cells in the G1 phase in the KD group was significantly higher than that in the NC group, and cells in the G2/M phase in the KD group was significantly fewer than that in the NC group, whereas the number of cells in the S phase was not significantly different between the KD group and the NC group (Fig. 5D). These experiments indicate that SCUBE3 promotes the proliferation of HCC cells, inhibits the apoptosis of HCC cells, and promotes the transformation of HCC cells from the G1 phase to the S phase. It was observed that CCNE1 had the same effect as SCUBE3 on the proliferation of HCC cells.

To further clarify whether SCUBE3 promotes the proliferation of HCC cells through the downstream molecule CCNE1, we overexpressed CCNE1 in SCUBE3 knockdown HCC cells. However, overexpression of CCNE1 in SCUBE3 knockdown cells did not affect the expression of SCUBE3, which suggested that the expression of CCNE1 might be regulated by SCUBE3 (Additional file 1: Figure S5).

**Fig. 6:**
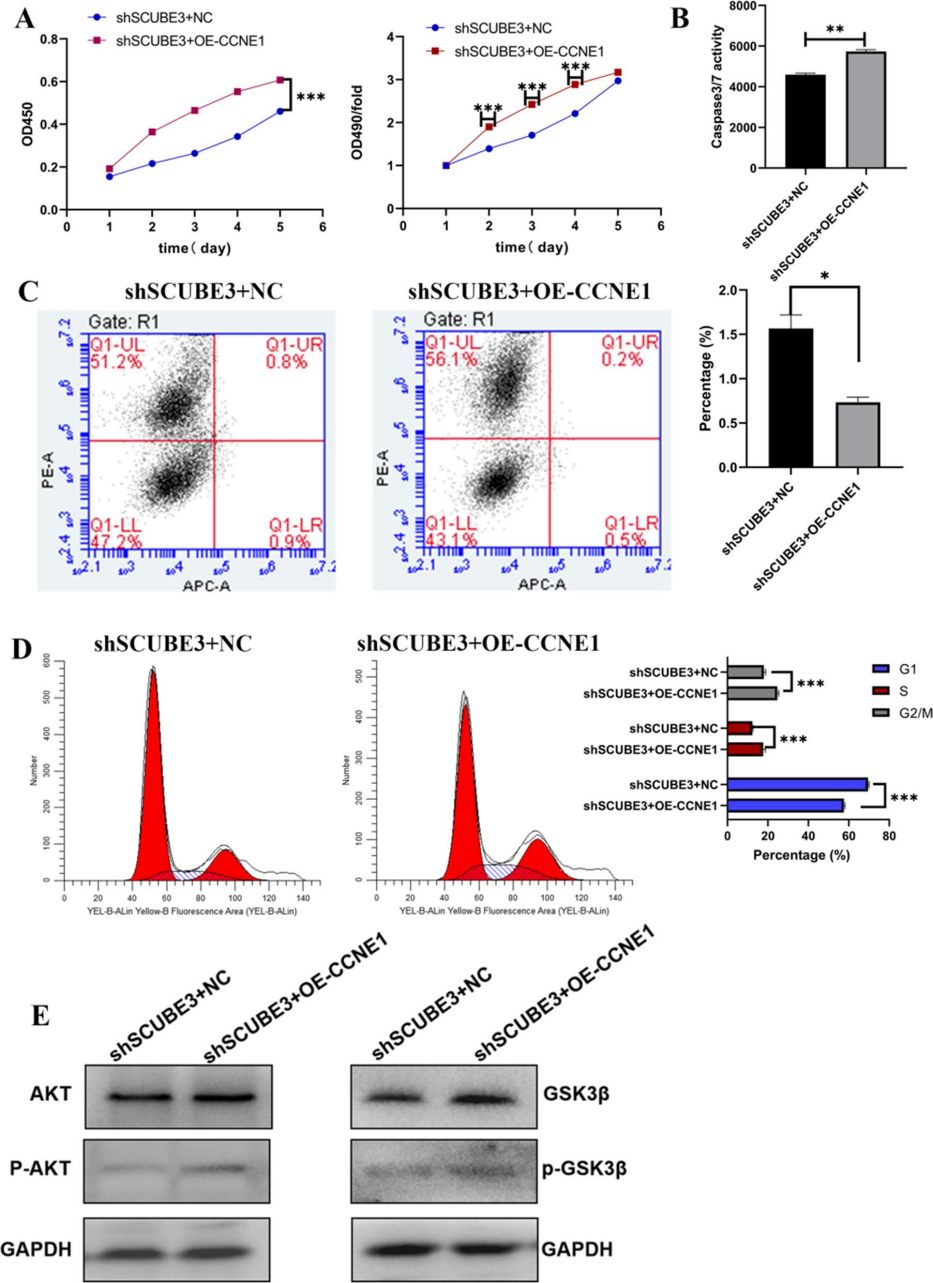
Overexpression of CCNE1 in SCUBE3 knockdown Bel7404 cells regulates proliferation and apoptosis. **A** MTT proliferation assay. **B** caspase 3/7 activity assay. **C** Detection of apoptosis in cells with both SCUBE3 knockdown and CCNE1 overexpression and control cells by the Annexin V-APC/PI double staining assay and, **D** cell cycle analysis of cells with both SCUBE3 knockdown and CCNE1 overexpression and control cells. **E** SCUBE3 knockdown cells were treated with overexpression lentivirus of CCNE1 for 72 h, Cell lysates were analyzed by immunoblotting using the indicated antibodies (*p < 0.01, **p < 0.01,***p < 0.001)

Next, we conducted MTT assay to verify whether overexpression of CCNE1 influences the effect of SCUBE3 knockdown on HCC cell proliferation. The proliferation of CCNE1-OE (overexpression) cells was significantly higher than that of cells in the NC group (Fig. 6A). The results of the two apoptosis detection experiments showed that CCNE1 overexpression reverses the influence of SCUBE3 knockdown on the apoptosis of liver cancer cells (Fig. 6B, C). Cell cycle analysis showed that the proportion of cells in the G1 phase was significantly lower in the CCNE1-OE group than in the NC group. Furthermore, we checked the expression level of downstream AKT/GSK3B in this rescue experiment, and found that the phosphorylation level of AKT/GSK3B was increased in cells of overexpressing CCNE1, but we did not observe that the total AKT/GSK3B protein expression level was not Variety (Fig. 6E). This indicated that CCNE1 overexpression reverses the influence of SCUBE3 knockdown on the cell cycle of HCC. Together, these findings demonstrated that SCUBE3 promotes proliferation of hepatoma cells through CCNE1.

### SCUBE3 inhibited the activity of GSK3 through the TGFβ non-classical PI3K/AKT pathway

**Fig. 7:**
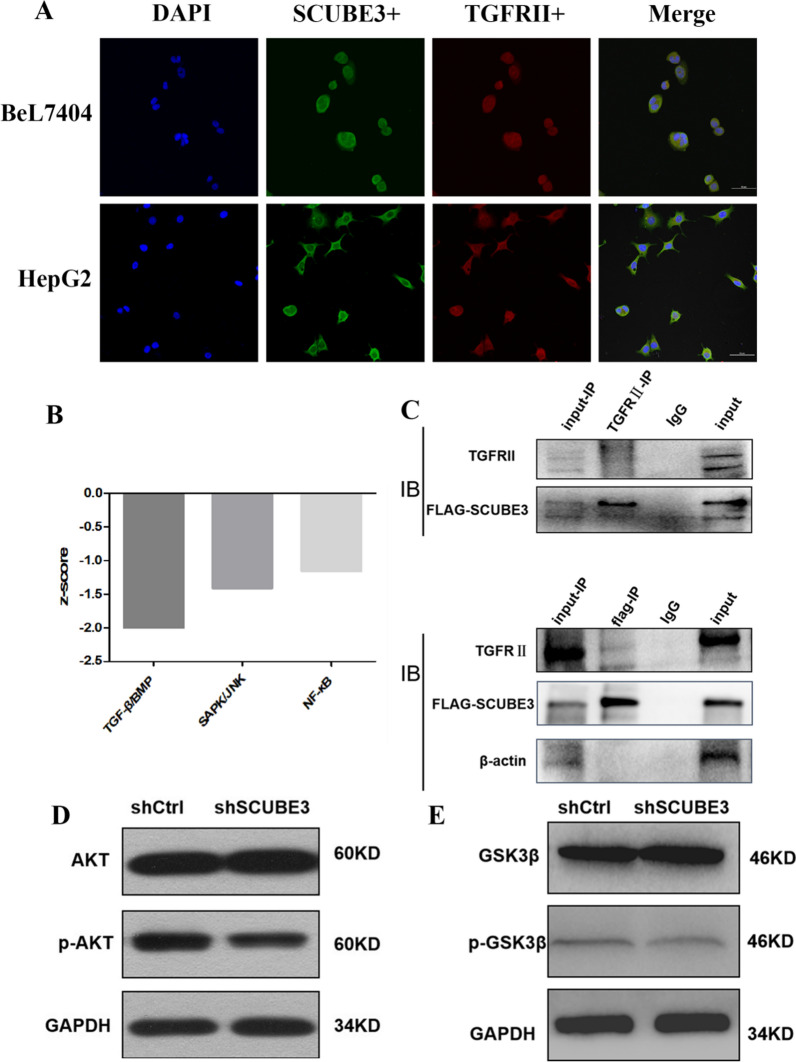
SCUBE3 binds to the TGFβIIR. **A** Co-localization of SCUBE3 and TGFβIIR. HepG2 and Bel7404 cells were stained with antibodies against SCUBE3 and TGFβIIR, followed by incubation with FITC-conjugated donkey anti-rat or anti-mouse IgG. The cells were visualized using a confocal microscope. The yellow areas represent protein co-localization. **B** The TGFβ pathway is significantly downregulated after SCUBE3 knockdown. **C** Co-immunoprecipitation assay results indicate that SCUBE3 interacts with TGFβIIR. **D** Western blot analysis of AKT and p-AKT expression in SCUBE3 knockdown cells and control cells. **E** Western blot analysis of GSK3β and p-GSK3β expression in SCUBE3 knockdown cells and control cells

To verify how SCUBE3 promotes the proliferation of liver cancer cells through CCNE1, we first applied KEGG enrichment analysis on the DEGs in the classical pathway screened by Affymetrix GeneChip microarray analysis (Fig. 7B). The TGFβ pathway was significantly downregulated. In non-small-cell lung cancer, SCUBE3 is reported to bind to type 2 TGFβ receptor (TGFβIIR) to activate the TGFβ pathway. Therefore, we used confocal microscopy to observe the subcellular localization of SCUBE3 and TGFβIIR after immunostaining. The results showed that SCUBE3 and TGFβIIR were co-located in the cytoplasm of HepG2 and Bel7404 cells (Fig. 7A). Then, we demonstrated the binding of SCUBE3 to TGFβIIR through co-immunoprecipitation analysis (Fig. 7C). While the TGFβreceptor can activate both the classical and non-classical SMAD-dependent pathways, including MAPK, PI3K/AKT, and rho-like GTPase signaling pathways, the PI3K/AKT pathway was also present among the ten most significantly downregulated pathways according to KEGG analysis. Therefore, we detected the expression of AKT and p-AKT in the SCUBE3-KD group and NC group by western blotting (Fig. 7D). The results showed that the expression of AKT was not significantly different between the two groups, whereas that of p-AKT was significantly higher in the NC group than in the KD group (Fig. 7D). This result indicates that PI3K/AKT is significantly inhibited after SCUBE3 knockdown and that SCUBE3 can phosphorylate AKT and activate the AKT pathway by activating TGFβIIR. CCNE1 is degraded through ubiquitination after its phosphorylation, and it can be phosphorylated by CDK2 and GSK3. AKT phosphorylation inhibits the kinase activity of GSK3. Therefore, we suspect that the lower activation of the AKT pathway in SCUBE3 knockdown cells would result in lower inhibition of GSK3 activity and consequently lower degradation of CCNE1, which leads to its accumulation. We detected the expression of GSK3 and p-GSK3 in SCUBE3-KD and NC cells by western blotting (Fig. 7E). The results showed that there was no significant difference in the expression of GSK3 between the KD group and the NC group, but the phosphorylation of GSK3 was significantly higher in the NC group than in the KD group, indicating that knockdown of SCUBE3 significantly reduced the phosphorylation of GSK3.

In summary, SCUBE3 inhibits the degradation of CCNE1 by activating the non-classical TGFβ pathway i.e., the PI3K/AKT pathway, to phosphorylate GSK3. CCNE1 is the key protein for cell transformation from the G1 phase to the S phase. Therefore, the accumulation of CCNE1 eventually promotes the transformation of liver cancer cells from the G1 phase to the S phase, promoting the proliferation of liver cancer cells.

## Discussion

HCC is the most common form of all types of liver cancer. Like other tumours, HCC cells have the characteristics of high proliferation and low apoptosis compared with normal cells. However, the molecular mechanism promoting the proliferation of HCC cells is not completely clear. Therefore, it is of great significance to study the pathogenesis of HCC, identify the key signaling pathways and essential regulatory proteins in the pathways, and improve the prognosis of patients with HCC.

Recently, the SCUBE family of genes and proteins has received considerable attention. These proteins play essential roles in several cellular functions, and they have been implicated in the pathogenesis of both neoplastic and non-neoplastic disorders. SCUBE3, an extracellular secretory protein of the SCUBE family, is located on cytokines and cell surfaces that play an essential role in auxiliary receptor function [15]. Early studies have found that SCUBE3 can participate in cardiac hypertrophy in mice through transcriptional activation mediated by TGFβ1 [16]. SCUBE3 also participates in the formation and development of mouse embryos [17]. In tumours [14, 20], SCUBE3 not only mediates the angiogenesis of lung cancer and promotes metastasis and invasion by promoting epithelial-mesenchymal transformation but also promotes the proliferation of osteosarcoma cells and influences the prognosis of patients. In a recent study [25], the expression of SCUBE3 in breast cancer cells and tissues increased significantly, and its expression was high in primary glioma specimens but low in normal brains [21]. However, the role of SCUBE3 in the proliferation of HCC cells is unclear. The Human Protein Atlas (https://www.proteinatlas.org/) shows that SCUBE3 expression is low in the liver. According to the analysis of the TCGA database at the UALCAN website, we found that SCUBE3 expression in HCC tissues is relatively increased, and there was a significant association between SCUBE3 expression and age, sex, differentiation grade, and clinical stage (Fig. 1A). However, we could not observe any obvious increasing trend in SCUBE3 expression with the progression of the disease. Therefore, it was selected as an essential candidate for further HCC research. First, we established a SCUBE3 knockdown cell model for cell proliferation, apoptosis, and cell cycle experiments in vitro (Fig. 1E–H). Our findings suggest that the downregulation of SCUBE3 remarkably suppresses the proliferation and induces apoptosis of HCC cells (Fig. 2A–E). Results of the first experiment suggested that SCUBE3 may affect the proliferation of HCC cells by arresting the cell cycle in the G1/S phase (Fig. 1F). The second experiment was conducted to evaluate the effect of SCUBE3 on the proliferation of HCC cells in vivo by establishing a subcutaneous implantation model in nude mice (Fig. 3). Our results show that SCUBE3 may be an oncogene for cell proliferation in Bel7404 cells in vivo and in vitro. However, to understand the functions of SCUBE3 in greater detail, its role should be examined in more cell lines.

SCUBE3 has been reported to modulate TGF-β, FGF, and hedgehog signaling [14, 26, 27]. In a study about chondrocyte proliferation and osteogenic differentiation, cell surface-bound SCUBE3 was found to be expressed as a BMP2/4 co-receptor in a cell-autonomous manner [22]. However, in the study of the proliferation of other tumours, the biological mechanism of SCUBE3 is not accurately described. Previous genomic, epigenomic, histopathological, and immunological analyses [28, 29] have confirmed the molecular drivers of HCC. The proliferation class among molecular classes accounts for ~ 50% of its biological activity [30]. To explore the underlying molecular biochemical mechanisms, 894 DEGs in SCUBE3-KD and SCUBE3-NC groups were screened by using Affymetrix GeneChip microarray analysis (Fig. 4).

Previous studies have shown that CDK6, CCND1 and CCNE1 play an important role in the proliferation of hepatoma cells [31–35]. We used western blotting to verify the protein expression level of the three possible downstream effectors (Fig. 4E). In the knockdown group, the downregulation of CCNE1 was the most obvious. CCNE1 is an activator of CDK2 and plays a key role in the regulation of mammalian cell cycle transition from the G1 phase to the S phase [36, 37]. Once the CCNE1 / CDK2 complex is formed, it will promote the phosphorylation of p27 and Rb proteins involved in the process of the cell cycle, replication factors A and C in DNA replication, and NPAT protein in histone biosynthesis so as to promote the transformation of cells from the G1 phase to the S phase, and finally promote cell proliferation [32, 38]. Some studies have consistently shown that CCNE1 is associated with disease progression in various malignancies and is clinically associated with poor prognosis in patients with ovarian, breast, bladder and colorectal cancer [39–42]. We also found that the use of RNA interference to target CCNE1 inhibits the neoplastic growth of a wide variety of liver cancers [43, 44]. We knocked down CCNE1 in HCC cells according to the molecular Koch's molecular postulates (Additional file 1: Figure S4). Although we did not observe a significant difference in wound healing between knockdown CCNE1 and control cells in the scratch experiment, the proliferation, apoptosis, and cell cycle analysis results were consistent with those obtained with SCUBE3 knockdown (Fig. 5). Overexpression of CCNE1 in SCUBE3 knockdown stable cell lines showed that it could reverse the effects of SCUBE3 knockdown on the proliferation, apoptosis, and cell cycle of HCC cells (Fig. 6). However, the SCUBE3 expression level was not changed in these CCNE1 overexpression cells. Therefore, SCUBE3 enhances the proliferation of HCC cells mainly through CCNE1, and the result suggests that CCNE1 is a downstream gene of SCUBE3. Enrichment analysis of the classical pathway and literature review showed that SCUBE3 might affect CCNE1 by binding with TGFβIIR to activate the non-classical pathway PI3K/AKT. The hypothesis was validated by immunoprecipitation and western blotting. However, the PI3K/AKT pathway does not directly affect CCNE1. CCNE1 is mainly degraded by ubiquitination through its own phosphorylation, which mainly occurs by the activity of GSK3β and CDK2 [45]. GSK3β can be phosphorylated by AKT in the PI3K/AKT pathway and inhibited by phosphorylation. Therefore, we suspect that the PI3K/AKT pathway inhibits the activity of CCNE1 kinase by phosphorylating GSK3β in HCC cells and then inhibits the ubiquitination of CCNE1, which eventually leads to the accumulation of CCNE1 and promotes the proliferation of HCC cells. To verify this hypothesis, we detected the expression and phosphorylation of GSK3β in SCUBE3-KD and SCUBE3-NC cells by western blotting. The results revealed no significant difference in the expression of GSK3β between the two groups, but the phosphorylation of GSK3β in the SCUBE3-KD group was significantly lower than that in the SCUBE3-NC group (Fig. 7).

However, the TGF signaling pathway is a complex signaling network. We have not studied the relationship between PI3K/AKT pathway and other pathways in depth. But this is the first study on the role of SCUBE3 in the proliferation of HCC cells, which provides a new direction for further study of the mechanism of HCC cell proliferation in the future and indicates that SCUBE3-targeting treatment may be promising in the therapies of patients with HCC. In addition, SCUBE3 may be a new molecular target for clinical diagnosis because of its secretory nature and a large number of clinical samples is still required for further confirmation. The expression of SCUBE3 and CCNE1 in the liver tissue was analyzed by bioinformatics due to the lack of liver tissue samples, but it was not validated in the experiment. At present, according to literature search, we have not found any relevant research reports on SCUBE3 compounds. We look forword to find potential compounds targeting SCUBE3, Which is our next step research plan.

## Conclusions

**Fig. 8:**
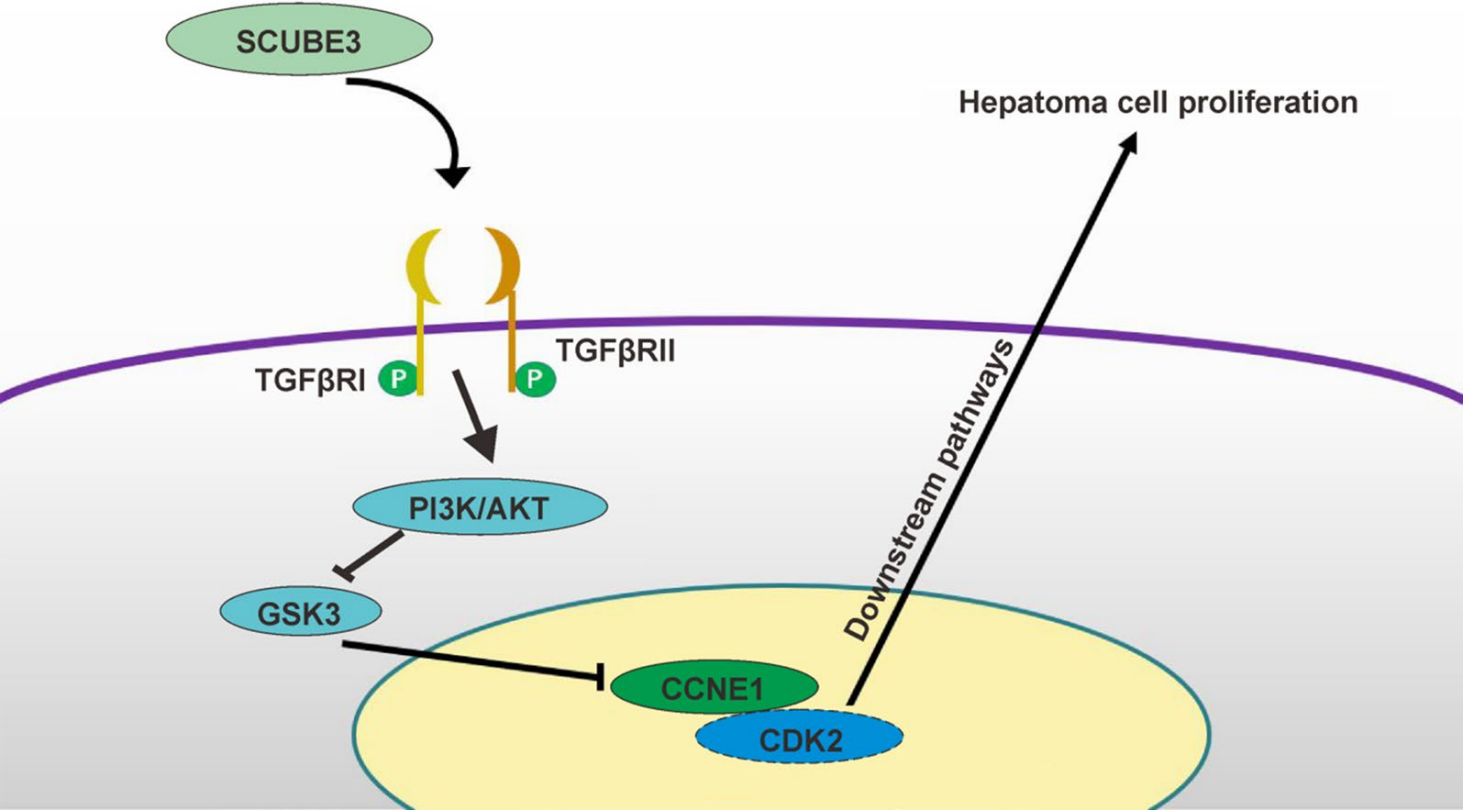
Schematic diagram of the validation hypothesis of this study: SCUBE3 activates the PI3K/AKT pathway by binding to the TGFβRII receptor to phosphorylate GSK3β to inhibit its kinase activity, thereby inhibiting the ubiquitination of CCNE1, leading to the accumulation of CCNE1 and ultimately promoting the proliferation of liver cancer cells

In conclusion, as shown in Fig. 8, this study mainly supports the hypothesis that SCUBE3 inhibits the activity of GSK3β kinase by activating the PI3K/AKT signaling pathway after binding to TGFβIIR, thereby inhibiting the degradation of CCNE1 and ultimately promoting the proliferation of HCC cells.

## Supplementary Information

**Additional file 1: Table S1. **The sequence of shRNA targeting human SCUBE3. **Table S2.** The sequence of shRNA targeting human CCNE1. **Table S3.** Primer sequences for quantitative RT-PCR. **Figure S1**. Quality evaluation of ChIP data revealed that the ChIP results were reliable. (A) Signal intensity distribution curve. (B) Pearson correlation coefficient distribution between samples. (C) Relative logarithmic signal strength box plot. (D) Principal component analysis score map. **Figure S2**. UALCAN expression analysis of CCNE1. (A) CCNE1 expression based on sample types. (B) CCNE1 expression based on patient's gender. (C) CCNE1 expression based on patient's age. (D) CCNE1 expression based on patient's weight. (E) CCNE1 expression based on tumour grade. (F) CCNE1 expression is based on individual cancer stages ***P < 0.001, ns,ns,no significance. **Figure S3.** UALCAN Survival Analysis of CCNE1. (A) Effect of CCNE1 expression level on survival of patients with hepatocellular Carcinoma. (B) Effect of CCNE1 expression level and tumour grade on survival of patients with hepatocellular Carcinoma. (C) Effect of CCNE1 expression level and gender on survival of patients with hepatocellular Carcinoma. (D) Effect of CCNE1 expression level and body weight on survival of patients with hepatocellular Carcinoma. (E) Effect of CCNE1 expression level and race on survival of patients with hepatocellular Carcinoma. **Figure S4.** Stable CCNE1 knockdown lines were established. (A–C) Verification of CCNE1 knockdown was confirmed in vivo by fluorescent microscopy Real-time PCR and Western blot Assay. *P < 0.05. **Figure S5**.Overexpression of CCNE1 in SCUBE3 knockdown Bel7404 cells (A) Fluorescence images after overexpression. (B–C)Overexpress efficiency were assessed by qRT-PCR and western blot. **P < 0.01. **Figure S6.** (A) Representative images of wound-healing assays and (B) quantification of wound closure (n = 10, ns, no significant).

## Data Availability

The data supporting the conclusions of this paper are included in the manuscript.
