# Supplementary material for: SCUBE3 downregulation modulates hepatocellular carcinoma by inhibiting CCNE1 via TGFβ/PI3K/AKT/GSK3β pathway
Source: Cancer Cell Int. 2022 Jan 3;22:1. doi: 10.1186/s12935-021-02402-z (PMC8725472; doi:10.1186/s12935-021-02402-z)
Supplement: Supplementary file 1 — Additional file 1: Table S1. The sequence of shRNA targeting human SCUBE3. Table S2. The sequence of shRNA targeting human CCNE1. Table S3. Primer sequences for quantitative RT-PCR. Figure S1. Quality evaluation of ChIP data revealed that the ChIP results were reliable. (A) Signal intensity distribution curve. (B) Pearson correlation coefficient distribution between samples. (C) Relative logarithmic signal strength box plot. (D) Principal component analysis score map. Figure S2. UALCAN expression analysis of CCNE1. (A) CCNE1 expression based on sample types. (B) CCNE1 expression based on patient's gender. (C) CCNE1 expression based on patient's age. (D) CCNE1 expression based on patient's weight. (E) CCNE1 expression based on tumour grade. (F) CCNE1 expression is based on individual cancer stages ***P < 0.001, ns,ns,no significance. Figure S3. UALCAN Survival Analysis of CCNE1. (A) Effect of CCNE1 expression level on survival of patients with hepatocellular Carcinoma. (B) Effect of CCNE1 expression level and tumour grade on survival of patients with hepatocellular Carcinoma. (C) Effect of CCNE1 expression level and gender on survival of patients with hepatocellular Carcinoma. (D) Effect of CCNE1 expression level and body weight on survival of patients with hepatocellular Carcinoma. (E) Effect of CCNE1 expression level and race on survival of patients with hepatocellular Carcinoma. Figure S4. Stable CCNE1 knockdown lines were established. (A–C) Verification of CCNE1 knockdown was confirmed in vivo by fluorescent microscopy Real-time PCR and Western blot Assay. *P < 0.05. Figure S5.Overexpression of CCNE1 in SCUBE3 knockdown Bel7404 cells (A) Fluorescence images after overexpression. (B–C)Overexpress efficiency were assessed by qRT-PCR and western blot. **P < 0.01. Figure S6. (A) Representative images of wound-healing assays and (B) quantification of wound closure (n = 10, ns, no significant). [file 12935_2021_2402_MOESM1_ESM.docx]

**Tables**

**Table S1.** The sequence of shRNA targeting human SCUBE3.

| **NO.** |  | **Target sequence** |  |
| --- | --- | --- | --- |
| shSCUBE3-1 |  | GTGCATCTGCAAGTCTGGCTA |  |
| shSCUBE3-2 |  | ATGCAGTTGCAAGAAAGGCTA |  |
| shSCUBE3-3 |  | AGGCTGCAGTGCTGTCCATTA |  |
| CON313 |  | TTCTCCGAACGTGTCACGT |  |

**Table S2.** The sequence of shRNA targeting human CCNE1.

| **NO.** |  | **Target sequence** |  |
| --- | --- | --- | --- |
| shCCNE-1 |  | CCTGGATGTTGACTGCCTT |  |
| shCCNE-2 |  | CCATTTGCCATGGTTATAA |  |
| shCCNE-3 |  | CCAGGAAGAGGAAGGCAAA |  |
| CON008 |  | TTCTCCGAACGTGTCACGT |  |

**Table S3.** Primer sequences for quantitative RT-PCR.

| **Gene** | **Primer sequences** |
| --- | --- |
| SCUBE3 | Forward: GAGTGCGAGCGAGAGATAATGC  Reverse: CCGTCATGTGCCAGGTGGAATC |
| CCNE1 | Forward: AGGTTTCAGGGTATCAGTGGTGC  Reverse: CTTTCTTTGCTCGGGCTTTGTCC |
| GAPDH | Forward: TGACTTCAACAGCGACACCCA  Reverse: CACCCTGTTGCTGTAGCCAAA |


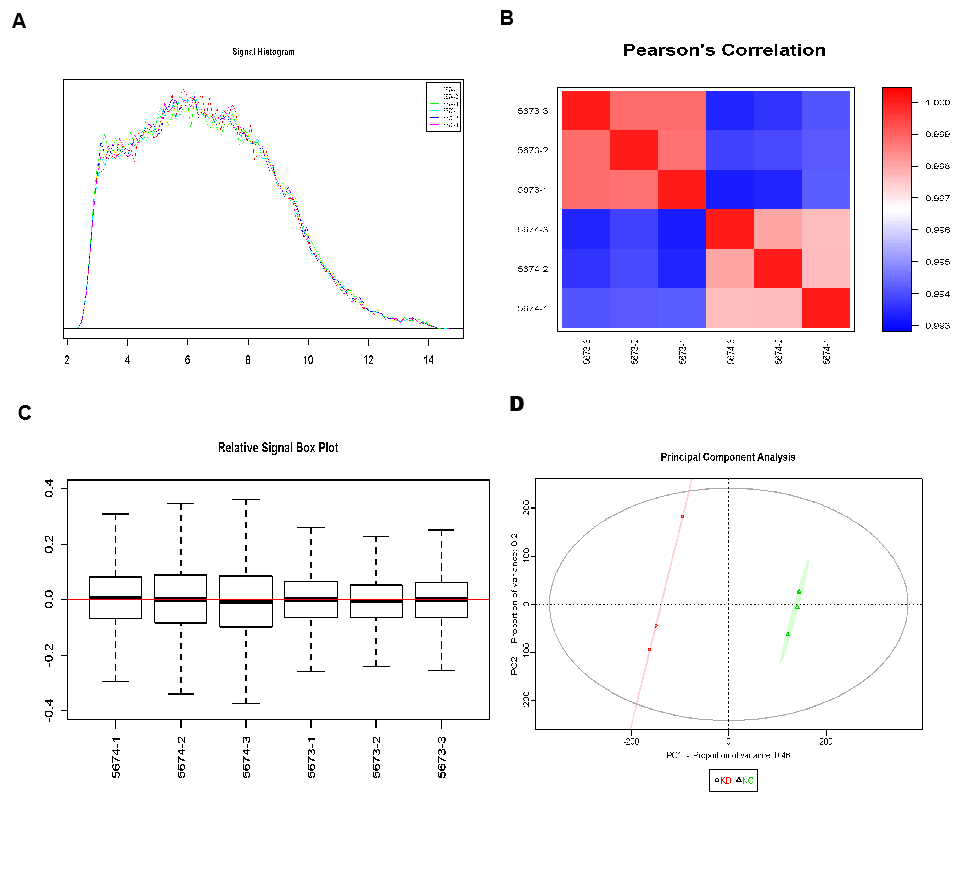


**Figure S1**. Quality evaluation of ChIP data revealed that the ChIP results were reliable. (A) Signal intensity distribution curve. (B) Pearson correlation coefficient distribution between samples. (C) Relative logarithmic signal strength box plot. (D) principal component analysis score map.


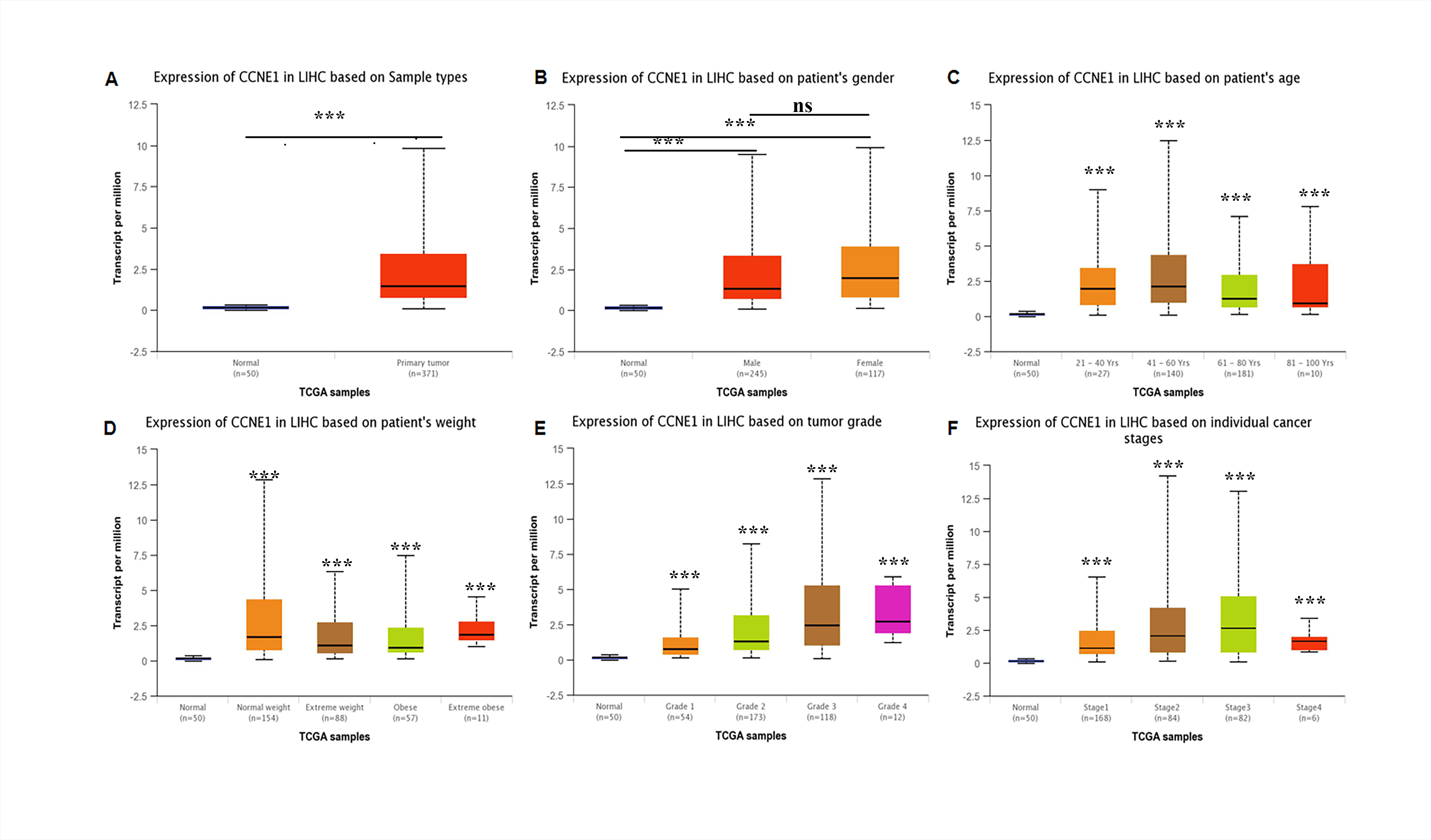


**Figure S2**. UALCAN expression analysis of CCNE1.(A) CCNE1 expression based on sample types. (B) CCNE1 expression based on patient's gender. (C)CCNE1 expression based on patient's age. (D) CCNE1 expression based on patient's weight. (E) CCNE1 expression based on tumour grade. (F) CCNE1 expression is based on individual cancer stages.***P < 0.001，ns,ns,no significance
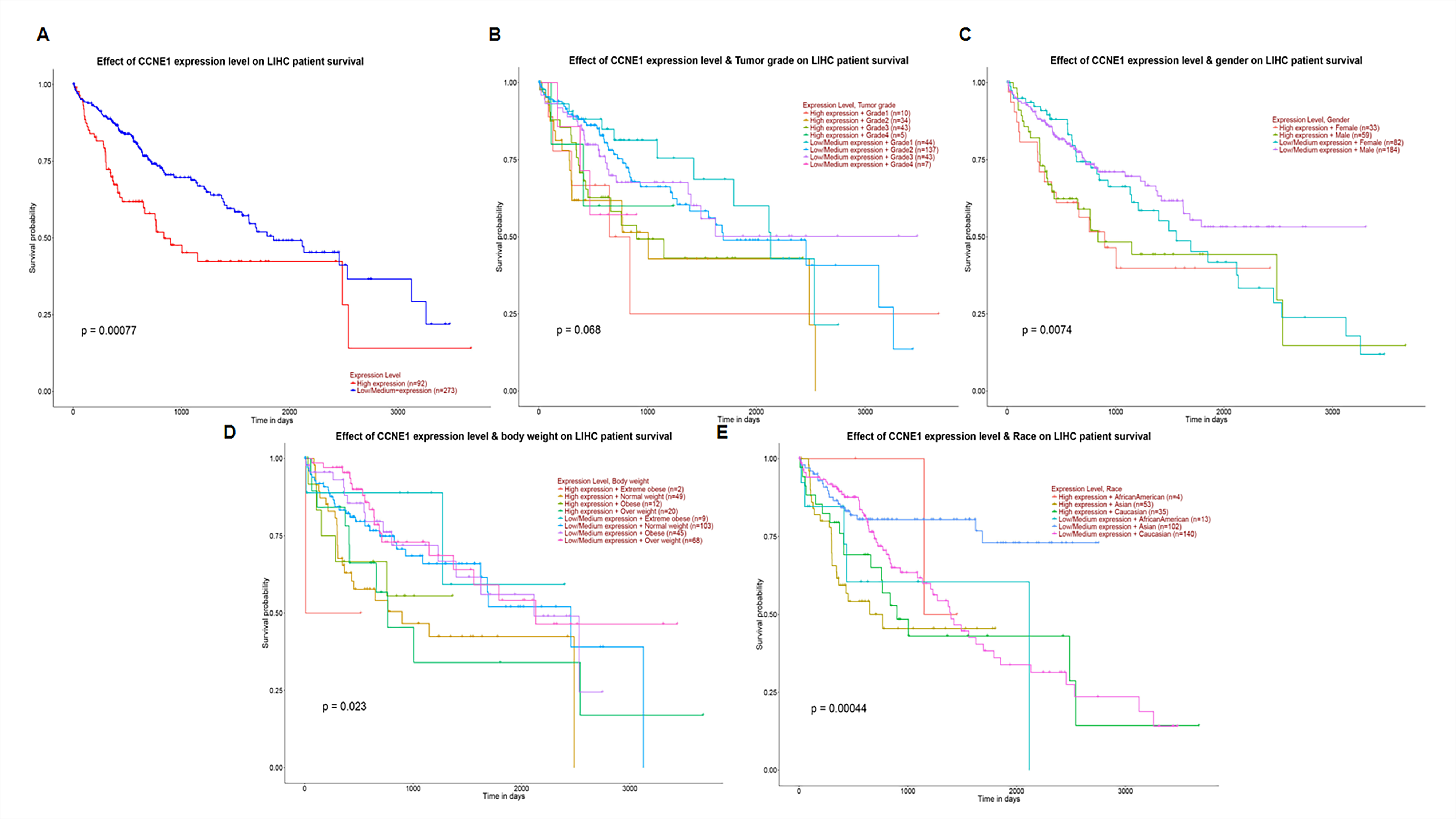


Figure S3. UALCAN Survival Analysis of CCNE1. (A) Effect of CCNE1 expression level on survival of patients with hepatocellular Carcinoma. (B) Effect of CCNE1 expression level and tumour grade on survival of patients with hepatocellular Carcinoma. (C) Effect of CCNE1 expression level and gender on survival of patients with hepatocellular Carcinoma. (D) Effect of CCNE1 expression level and body weight on survival of patients with hepatocellular Carcinoma. (E) Effect of CCNE1 expression level and race on survival of patients with hepatocellular Carcinoma.

Figure S4.Stable CCNE1 knockdown lines were established. (A-C) Verification of CCNE1 knockdown was confirmed in vivo by fluorescent microscopy Real-time PCR and Western blot Assay. *P< 0.05.
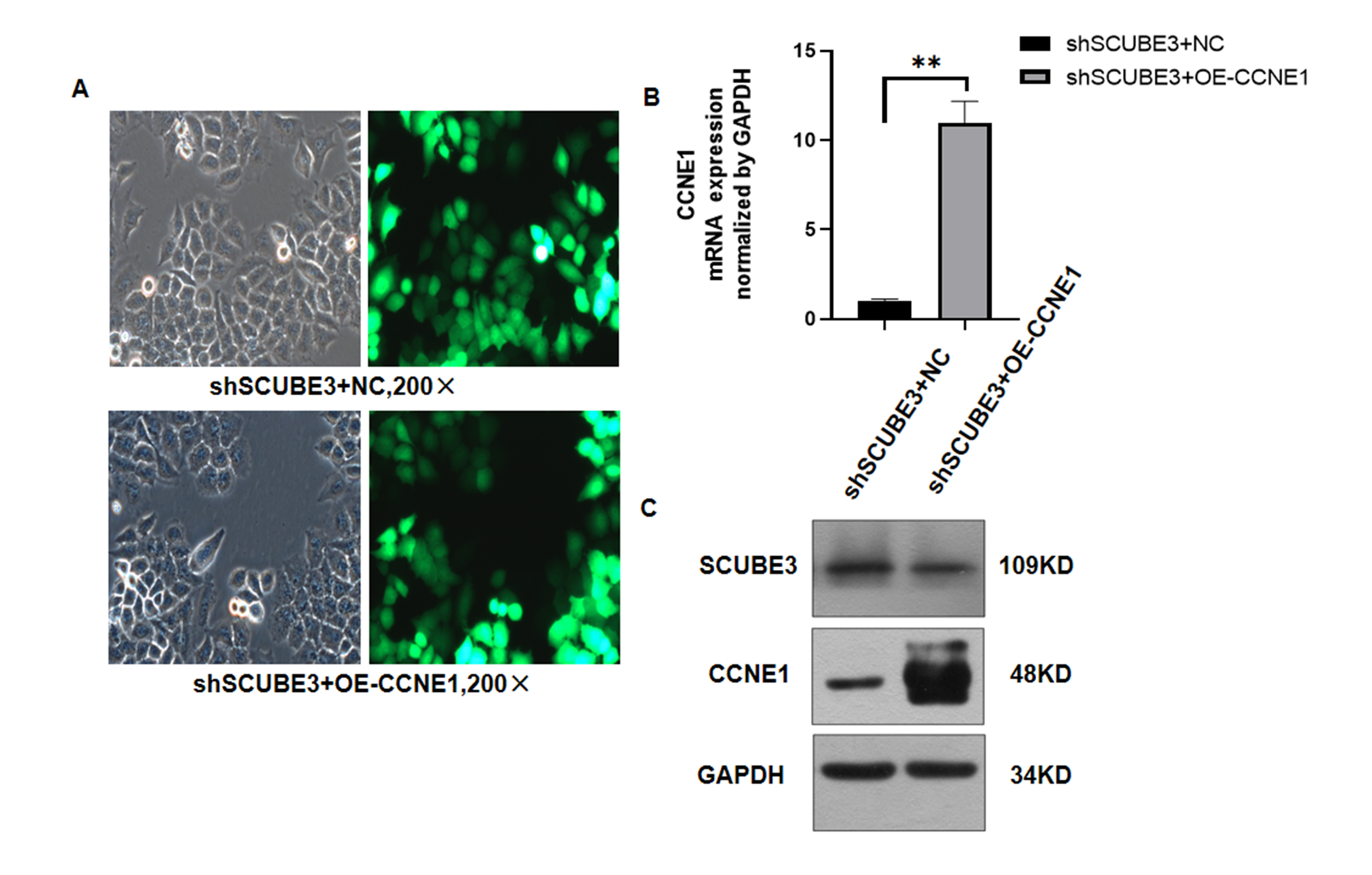


**Figure S5**. Overexpression of CCNE1 in SCUBE3 knockdown Bel7404 cells (A) Fluorescence images after overexpression. (B-C) Overexpress efficiency were assessed by qRT-PCR and western blot. **P < 0.01


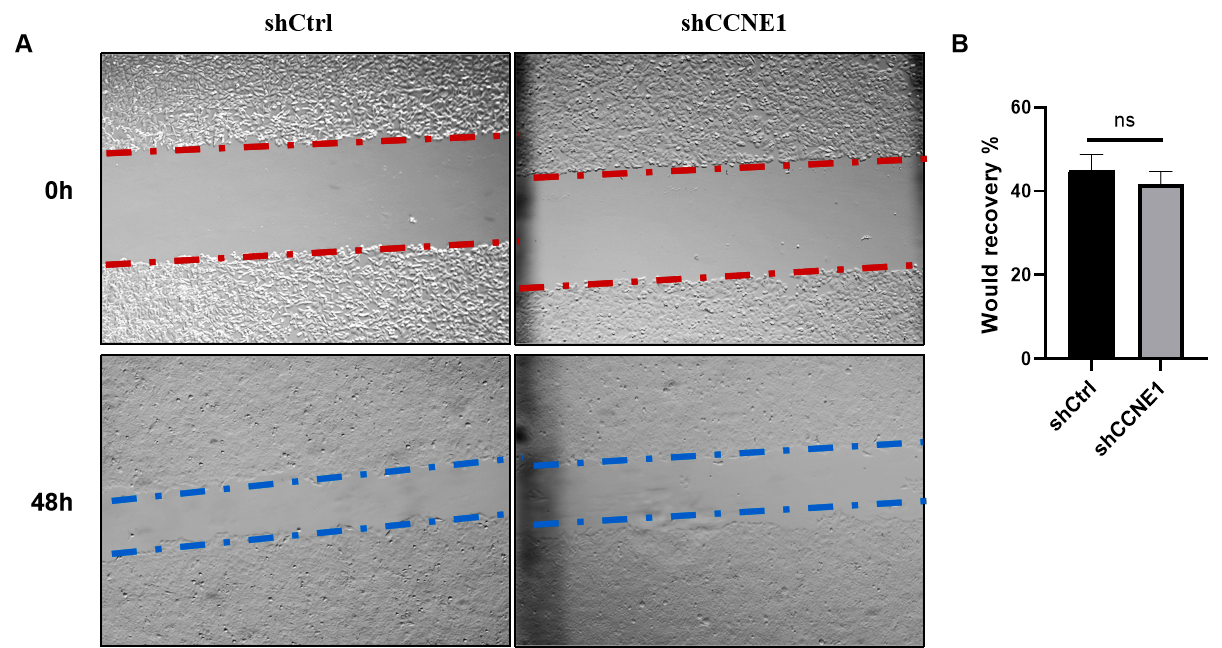


Figure S6.(A) Representative images of wound-healing assays and

(B ) quantification of wound closure. (n = 10, ns, no significant)
